# The Left Atrial Appendage: Anatomy, Electrophysiology, and Clinical Implications for Atrial Fibrillation and Stroke

**DOI:** 10.1007/s11886-026-02428-7

**Published:** 2026-10-06

**Authors:** Adriana Daniela Sârb, Csilla-Andrea Eötvös, Teodora Avram, Iulia-Georgiana Zehan, Mădălina Patricia Moldovan, Roxana Daiana Lazar, Giorgia Coșeriu, Patricia Schiop-Tentea, Ioana-Diana Mihuș, Oliviu Florențiu Sârb, Mihnea Istrate, Doinita Crisan, Edwin Kevin Heist, Sorin Pop, Dan Blendea

**Affiliations:** 1 Iuliu Hatieganu University of Medicine and Pharmacy, Cluj-Napoca, Romania; 2 Niculae Stancioiu Heart Institute, Cluj-Napoca, Romania; 3 Department of Infectious Diseases, Iuliu Hatieganu University of Medicine and Pharmacy, Cluj-Napoca, Romania; 4 Department of Pathology County Emergency Hospital, Iuliu Hatieganu University of Medicine and Pharmacy, Cluj-Napoca, Romania; 5 Massachusetts General Hospital, Harvard Medical School, Boston, MA 02114 USA; 6 Emergency Clinical County Hospital, Iuliu Hatieganu University of Medicine and Pharmacy, Cluj-Napoca, Romania; 7 Department of Internal Medicine, Iuliu Hatieganu University of Medicine and Pharmacy, Cluj-Napoca, Romania

**Keywords:** Left atrial appendage (LAA), Atrial fibrillation (AF), Electrical isolation, Stroke prevention, Antithrombotic therapy, LAA closure

## Abstract

**Purpose of Review:**

The left atrial appendage (LAA) is nowadays recognized as both an arrhythmogenic substrate and the dominant source of thromboembolism in atrial fibrillation (AF). In this review we tried to summarize recent data on the anatomy, physiology, and imaging of the LAA, and examine how these findings help guide rhythm-control and stroke-prevention strategies.

**Recent Findings:**

High-resolution mapping confirms the LAA as a non–pulmonary-vein trigger source in persistent and post-ablation AF, supporting LAA electrical isolation in selected patients, though isolation may induce mechanical standstill and mandate continued thromboprophylaxis. The 2024 ESC guidelines reframed stroke-risk assessment around the sex-neutral CHA₂DS₂-VA score within the AF-CARE pathway. An important recent study was the OPTION trial, which showed that LAA closure after AF ablation was as effective as oral anticoagulation for preventing death, stroke, or systemic embolism and was associated with less non–procedure-related bleeding. These findings support the use of LAA closure in selected patients after ablation. New imaging and emerging computational tools may also improve patient selection and help guide device sizing.

**Summary:**

A comprehensive assessment of the anatomy, function, and electrical activity of the LAA can help adapt AF treatment to each patient.

## Introduction

The left atrial appendage (LAA) was considered a vestigial structure contributing mainly to thromboembolic risk in atrial fibrillation (AF) [1]. Evidence now shows that the LAA is electrically and mechanically active and can initiate and sustain AF [2]. Understanding these roles of the LAA is essential as rhythm-control strategies now include LAA-targeted interventions [3].

Although AF affects more than 50 million people worldwide, the LAA’s role in arrhythmogenesis and stroke risk is not well defined [4]. The large majority of thrombi in nonvalvular AF arise from the LAA, however guidelines provide limited direction on which patients should receive appendage-targeted therapy.

We included in this review literature from 2000 to 2025 identified through PubMed and Scopus searches using relevant AF- and LAA-related terms. We prioritized electrophysiologic and interventional studies.

We tried to offer anatomical, functional, and procedural perspectives for the practicing cardiologist and electrophysiologist, focusing on how the LAA influences rhythm-control strategies and stroke prevention in contemporary AF management.

## Development and Anatomy of the LAA: Structural Basis for Arrhythmogenicity

**Fig. 1:**
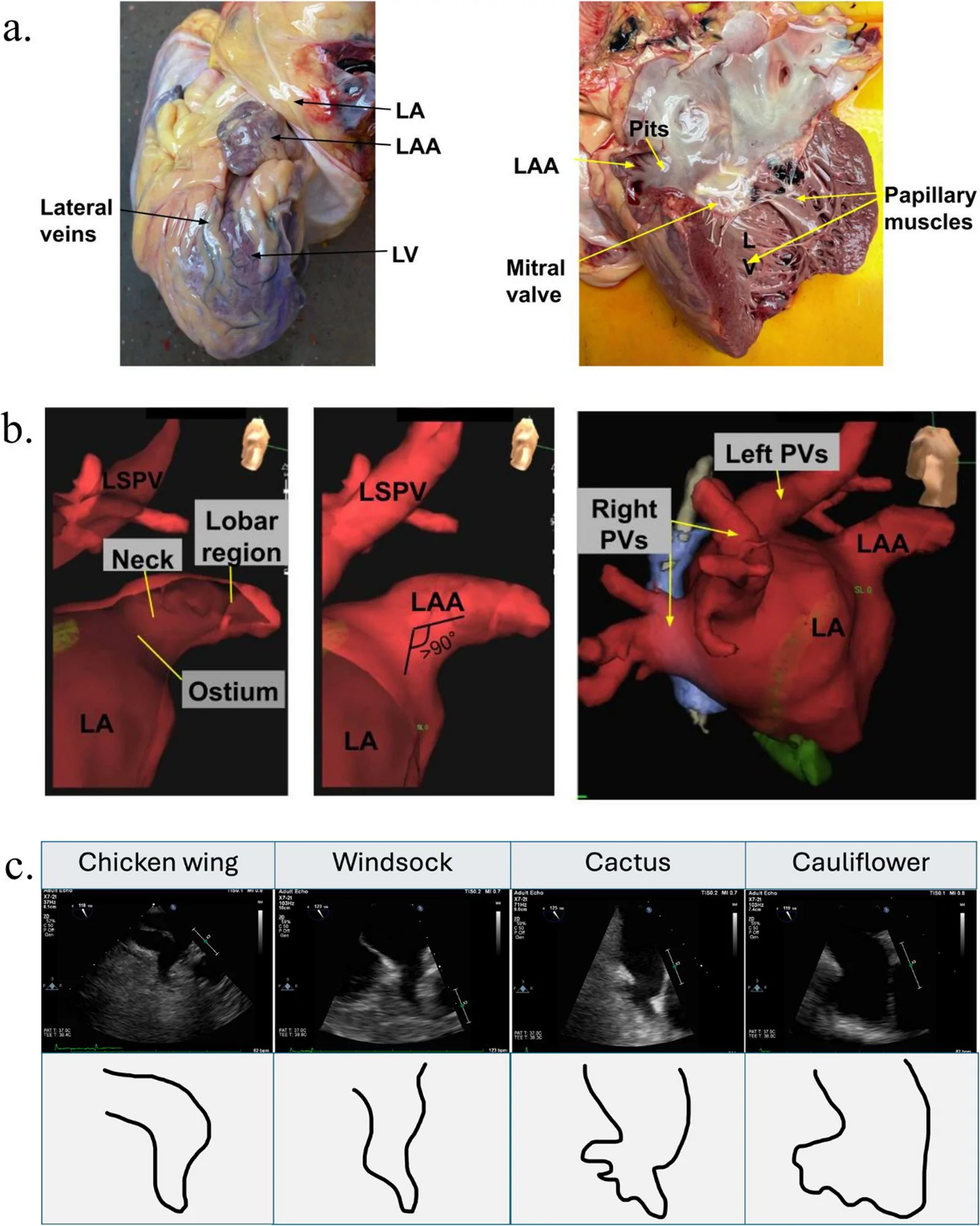
Anatomy, morphology, and clinical decision-making for the left atrial appendage. (**a**) Gross anatomic view of the LAA showing its relation to the left atrium (LA), left ventricle (LV), pulmonary veins (PVs), and mitral valve (MV); original figure created by the authors. (**b**) Three-dimensional reconstruction illustrating LAA lobar configuration and angulation. (**c**) The four principal LAA morphologies seen on transesophageal echocardiography. Key: LA left atrium, LAA left atrial appendage, LSPV left superior pulmonary vein, LV left ventricle, MV mitral valve, PVs pulmonary veins, TEE transesophageal echocardiography

The LAA is a trabeculated, finger-like extension of the primordial left atrium situated along the anterior atrial wall adjacent to the pulmonary artery (Fig. 1a) [5].

Several neighboring anatomical structures, like the phrenic nerve, mitral valve, left circumflex artery, and the left superior pulmonary vein, are important because they could be potentially injured during procedures targeting the LAA [5].

Embryologically, the LAA originates from the primordial left atrium and its wall has pectinate musculature, whereas much of the smooth-walled left atrial body develops through incorporation of the pulmonary veins. The LAA is also richly supplied by autonomic nerve fibers. This can explain at least in part the appendage’s unique contractile and conduction characteristics [6].

The complex muscular geometry of the LAA supports anisotropic conduction and local re-entry, helping sustain AF [7].

The anatomical components of the LAA include an ostium, short neck, and one or more lobes. One characteristic is its marked morphological variability (Fig. 1c) [7].

The LAA has between one and four lobes, most commonly two. A more complex structure of the LAA lobes was shown to correlate with slower appendage emptying and higher thrombogenic risk [7]. The shape of the LAA ostium is usually elliptical with the long axis diameter of 10–40 mm and the short axis of 8–30 mm. LAA depth varies from approximately 16–51 mm, with chicken-wing morphologies typically being shorter and more angulated [8]. These dimensions have an impact on closure-device sizing.

A widely used classification by Di Biase et al. describes four major shapes, with prevalences from the original 932-patient cohort [9, 10]:

- Chicken Wing – 48%.
- Cactus – 30%.
- Windsock – 19%.
- Cauliflower – 3%.

This study also revealed that the chicken-wing morphology was associated with a lower probability of prior stroke/TIA (odds ratio 0.21) independent of the CHADS₂ score. Cactus, windsock, and cauliflower morphologies carried approximately four- to eight-fold higher odds [10]. A subsequent meta-analysis confirmed a lower thromboembolic risk with chicken-wing when compared to non–chicken-wing morphology (OR 0.46) [1]. However, this four-type classification is subjective, shows limited inter-observer reproducibility, and has not yielded a validated prediction model. Chicken-wing morphology usually demonstrates proximal folding and a dominant lobe, permitting more efficient emptying. The LAAs displaying cactus, windsock, and cauliflower morphology typically have wider ostia, multiple lobes, and greater curvature, and are associated with higher thromboembolic and arrhythmogenic risk [7, 9, 11]. These geometric features can be characterized by transesophageal echocardiography (TEE) or computed tomography (CT) (Fig. 1c).

A complementary simplified system classifies LAA anatomy using the angle of the bend of the LAA. Appendages fall into two categories: <90° (lower-risk) or > 90° (higher-risk) (Fig. 1b) [7].

This system was proposed because of the limited reproducibility of the four-type classification, and it has the advantage of simplifying pre-procedural assessment of the LAA. Several anatomic properties of the appendage can have an impact on interventions. LAA wall thickness ranges 1–3 mm and is thinnest at the apex and distal lobes while trabeculation density is greatest near the ostium. These characteristics may influence the choice of power settings during the ablation procedure as well as the risk of perforation. It is worth highlighting also several other observations regarding LAA characteristics and their potential role in the peri-interventional setting, as reported in several studies. In one study, the chicken wing LAA morphology was associated with a significantly increased risk of peri-procedural thromboembolic events in patients undergoing atrial fibrillation ablation, with an approximately seven-fold higher risk compared with other LAA morphologies [12]. This contrasts with the lower long-term risk of chicken-wing appendages and likely reflects a different mechanism: catheter manipulation rather than chronic stasis.

Despite these findings, another study reported that late thromboembolic events following AF ablation were not associated with LAA morphology, but rather with AF recurrence (HR 6.2) and LAA orientation. A superior LAA takeoff was independently associated with an increased risk of thromboembolic events (HR 4.9) [13]. This association was also observed in patients with a low CHA₂DS₂-VASc score and a history of stroke, in whom a superior LAA takeoff was the only predictor of thromboembolic events (OR 9.1), while lower LAA flow velocities were associated with higher event rates. These findings led to the hypothesis that a higher LAA takeoff may be associated with tachycardia-mediated alterations in thrombogenic flow and, consequently, an increased risk of thromboembolic events [14].

LAA morphology-related considerations during implant of occlusion devices include secure device anchoring in chicken-wing appendages, the need for larger devices in windsock morphologies, and a higher rate of peri-device leak in cauliflower types.

Peri-procedural imaging using CT or intracardiac echocardiography (ICE) may define ostial orientation, depth, and neighboring vascular structures and guide safe access and LAA occlusion device deployment [5, 9].

In summary, the embryologic origin of the LAA contributes to its specialized muscular structure and marked morphological variability. These features explain both its thromboembolic risk and its importance during interventional planning. The impact of the anatomy on electrophysiologic properties is discussed in Sect. 5.

## Functional and Electrophysiologic Properties of the LAA

The LAA role is that of a reservoir that modulates left-atrial pressure and volume. Its filling occurs during ventricular systole and its emptying during diastole. The contraction of the LAA contributes to the LA booster-pump function and late-diastolic ventricular filling. The appendage has greater contractility and distensibility than the LA body. It acts as a decompression chamber if the LA pressure increases [9]. In sinus rhythm, LAA emptying velocities typically are in the vicinity of 50–60 cm/s. During AF, these decrease significantly. Emptying velocities lower than 20 cm/s are associated with stasis, spontaneous echo contrast, and increased thrombogenicity [9].

In addition to its mechanical role, the appendage has significant electrical activity. The latter is made possible by the dense autonomic innervation, heterogeneous fiber orientation, and variable distribution of the gap junctions. These features may facilitate triggered activity and localized re-entry. The LAA, as well as other adjacent structures such as the ligament of Marshall, represent important sources of non–pulmonary-vein triggers, particularly in persistent or post-ablation AF [15, 16].

Another role of the LAA is to contribute to neurohumoral regulation by secreting atrial natriuretic peptides. This role may be disturbed after procedures that exclude the LAA, with subsequent impact on atrial compliance and volume homeostasis, although the long-term effects remain uncertain [17].

## Imaging Evaluation of the LAA

Accurate imaging of the LAA is important for electrophysiologic planning before and for monitoring during ablation or occlusion procedures. The most important goals are detailed definition of LAA anatomy and thrombus detection [9]. Among different imaging modalities, TEE remains the gold standard for thrombus detection and is being used for real-time device deployment. Multidetector computed tomography (MDCT) provides excellent anatomic definition for morphology classification and device sizing [18]. Another imaging modality is cardiovascular magnetic resonance (CMR) which provides radiation-free functional assessment when iodinated contrast is contraindicated. ICE supports real-time intraprocedural imaging without the need for general anesthesia [19]. The main characteristics of each modality are summarized in Table 1.

**Table 1:** LAA imaging techniques

Modality	Key strengths	Key limitations	Primary clinical use
Transesophageal Echocardiography (TEE)	- Gold standard for thrombus detection. - Real-time visualization. - Assesses LAA flow velocity. − 3D for sizing.	- Semi-invasive. - Requires sedation. - Contraindicated in esophageal disorders. - Operator-dependent.	- Thrombus exclusion. - Guidance during closure.
Multidetector Computed Tomography (MDCT)	- Excellent anatomic detail. - Morphology classification. - Device sizing. − 3D reconstruction.	- Radiation and iodine contrast exposure. - Lower temporal resolution.	- Pre-procedural planning and follow-up.
Cardiovascular Magnetic Resonance (CMR)	- No radiation. - Functional and tissue characterization. - Thrombus detection.	- Long acquisition time. - Motion sensitivity. - Contraindications with some implants. - Less anatomic detail than CT.	- Alternative when contrast or radiation are contraindicated.
Intracardiac Echocardiography (ICE)	- Real-time intraprocedural imaging. - Transseptal guidance. - Reduced fluoroscopy. - Avoids general anesthesia.	- Invasive. - Limited field of view. - Catheter cost. - Operator-dependent.	- Guidance during ablation and closure.

Clinical indication for the procedure and patient characteristics are helpful when trying to choose a particular imaging modality. TEE is preferred in order to exclude thrombus before cardioversion and LAA occlusion. MDCT is usually limited to the pre-procedural stage for defining dimensions and morphology subtype [18]. ICE is increasingly used during ablation and closure procedures in patients who cannot undergo TEE [20]. CMR can be useful when trying to avoid radiation or use of iodinated contrast, particularly in younger patients or those with renal dysfunction.

These imaging techniques can be combined in a multimodal approach, especially during ablation procedures. Pre-procedural CT or CMR datasets can be integrated with electroanatomic mapping to increase the accuracy and safety of lesion placement during ablation [21]. Post-procedural imaging, especially TEE, can be used to assess peri-device leak and device-related thrombus. Residual leak is common (roughly 10–30% on TEE, more on CT), while device-related thrombus occurs in about 2–4% [22].

## Role of the LAA in AF

The LAA is a complex, electrically active structure. There are several reasons related to its anatomy and pathophysiology which could explain its arrhythmogenic potential. Its pectinate muscles can have a heterogeneous architecture. This creates the substrate for slow, anisotropic conduction and micro-reentry. Another mechanism for LAA arrhythmias is represented by triggered activity caused by mechanical stretch in the setting of increased intraatrial pressure. The dense sympathetic and parasympathetic innervation of the LAA modulates refractoriness and can further increase the spatial dispersion of action-potential duration. These mechanisms create conditions for both focal automaticity and reentry. The arrhythmia substrate in the LAA seems to resemble that described by high-resolution optical mapping in the pulmonary veins [23].

Autonomic activation lowers LAA effective refractory period more than in the main LA body, promoting conduction heterogeneity [24].

Di Biase et al. demonstrated that non-pulmonary-vein triggers originate from the LAA in up to 27% of patients with recurrent AF after pulmonary vein isolation. Focal activity most frequently is located at the LAA ridge or within the proximal lobes. Ablation of these sites frequently terminates or slows AF, confirming their role in the arrhythmia mechanism [2].

The role of the LAA in AF pathogenesis appears to vary with arrhythmia duration. In patients with paroxysmal AF, the LAA generally plays a limited role, whereas in persistent AF, LAA-related triggers become more prominent [25].

Given the role of the LAA as a non-pulmonary vein trigger for AF, electrical isolation of the LAA by circumferential ablation around the ostium has been investigated as an adjunctive strategy for refractory, non-paroxysmal AF.

The randomized BELIEF trial reported improved freedom from arrhythmia when empirical LAA isolation was added to extensive left-atrial ablation in long-standing persistent AF (56% vs. 28% freedom from arrhythmia after a single procedure at 12 months, and 76% vs. 56% cumulative success at 24 months after a mean of 1.3 procedures). This strategy however, remains largely confined to experienced centers, and results across groups have been inconsistent [26, 27].

In addition, isolation renders the appendage akinetic and substantially increases thromboembolic risk. In the largest series reported to date, stroke or transient ischemic attack occurred in 16.7% of patients with impaired post-isolation LAA contractility who discontinued anticoagulation, compared with 1.7% of those maintained on uninterrupted therapy [3]. No strokes occurred in patients whose LAA contractility was preserved after isolation. Uninterrupted anticoagulation is therefore mandatory, and concomitant percutaneous LAA closure has been proposed to mitigate this iatrogenic risk.

The LAA wall is thin and variable in thickness, necessitating careful energy titration to avoid perforation or pericardial effusion. Contact-force and temperature monitoring, together with intracardiac echocardiographic visualization of lesion formation, improve procedural safety [21]. Incomplete isolation predisposes to reconnection and arrhythmia recurrence, whereas excessive lesion delivery risks collateral injury to the circumflex artery or left phrenic nerve. Accordingly, LAA isolation is generally reserved for specialized centers with experience in complex left-atrial ablation.

Recognition of the LAA as an arrhythmogenic source supports an individualized approach to advanced AF ablation. Mapping-guided identification of LAA triggers, particularly in redo and persistent cases, can refine lesion strategy. Pre-procedural CT or intracardiac echocardiography helps define ostial orientation and wall thickness to facilitate targeted, transmural lesion delivery. In carefully selected patients, combining LAA isolation with simultaneous mechanical closure may allow rhythm stabilization and stroke prevention within a single intervention. Trials of contemporary occluder devices are informing long-term outcomes and anticoagulation strategies following mechanical exclusion [28].

The LAA thus represents a major non-pulmonary-vein contributor to AF maintenance, particularly as the disease progresses toward persistent forms.

## Thrombosis and Stroke Risk in the Context of Rhythm Management

Atrial fibrillation management often requires addressing symptoms and stroke prevention at the same time. Symptom relief can be achieved through rhythm control, a strategy often preferred in younger patients and in those with persistent symptoms or heart failure [29, 30]. Restoration of sinus rhythm alleviates symptoms but does not eliminate the thromboembolic risk associated with AF. Therefore, stroke prevention remains an essential part of care not only in patients undergoing rhythm control but in all patients with AF. Changes involving the LAA structure and hemodynamics in AF create conditions for thrombus formation. Atrial dilation and fibrosis impair LAA function, leading to slower blood flow, and contribute to the arrhythmogenic substrate and create a thrombogenic milieu [31]. Electrical isolation of the appendage may increase the thrombosis risk further by reducing or abolishing LAA contractility [3].

### Oral Anticoagulation

Oral anticoagulation (OAC) remains the cornerstone of stroke prevention in AF. The indications for OAC are based on the estimated thromboembolic risk. While the 2023 ACC/AHA/ACCP/HRS guideline uses for this purpose the CHA₂DS₂-VASc score, the 2024 ESC guidelines simplified risk assessment by replacing CHA₂DS₂-VASc with the CHA₂DS₂-VA score, which excludes sex category [30, 32]. The ESC guideline recommends OAC for patients with a score ≥ 2 and consideration of OAC for those with a score of 1. Vitamin K antagonists reduce stroke risk by approximately two-thirds when the INR is maintained between 2.0 and 3.0 whereas direct oral anticoagulants, including dabigatran, rivaroxaban, apixaban, and edoxaban, provide comparable or greater efficacy with a more favorable safety profile. Despite this, between 10 and 15% of patients carry contraindications to long-term anticoagulation [33–36]. For these patients, LAA closure has emerged as an important alternative to long-term anticoagulation.

### LAA Closure for Stroke Prevention

For patients unable to take long-term anticoagulation, mechanical exclusion of the LAA provides an effective solution to reduce the thromboembolic risk in AF (Table 2).

**Table 2:** LAA closure techniques (percutaneous vs. surgical) with reported success and complication rates

Approach	Example techniques	Success rate	Major complications	Typical role
Percutaneous	*WATCHMAN*,* Amulet*,* LAmbre*	− 96% [37]	2–3% (effusion, embolization, peri-device leak, device-related thrombus) [38];	Patients unsuitable for long-term OAC or post-LAA isolation
Surgical	*AtriClip*,* excision*	87–98% [39, 40]	< 1% when concomitant [39]	Performed during cardiac surgery

One approach to LAA exclusion is by percutaneous closure. This is performed via transseptal access under TEE or ICE guidance and achieves procedural success in approximately 96% of cases [37, 41]. Currently used devices include plug-type occluders such as the WATCHMAN and Amulet, as well as lobe-filling systems. The device is selected using ostial morphology and landing-zone anatomy as guiding factors [19, 42]. After the LAA occlusion procedure TEE is used to confirm seal integrity and exclude device-related thrombus [22].

Major complications, predominantly pericardial effusion and device embolization, occur in approximately 2.5 to 4.5% of procedures. In the randomized Amulet IDE trial these occurred in 2.5% of patients treated with the WATCHMAN and in 4.5% of those treated with the Amulet [28]. An alternative approach is surgical LAA exclusion. This is most commonly performed concomitantly with planned cardiac surgery, with success rates ranging from 87 to 98% for clipping and excision and complication rates below 1% [39, 40].

The randomized OPTION trial provided the first head-to-head comparison of LAA closure with contemporary oral anticoagulation in patients undergoing AF ablation. Among 1,600 patients with elevated thromboembolic risk, closure with a WATCHMAN FLX device was non-inferior to continued anticoagulation for the composite of death from any cause, stroke, or systemic embolism at 36 months (5.3% vs. 5.8%), and superior for non–procedure-related major or clinically relevant non-major bleeding (8.5% vs. 18.1%). Of note, 95% of the comparator arm received a direct oral anticoagulant. The OPTION trial enrolled patients undergoing ablation, not LAA isolation, so it does not directly test the combined strategy [43].

### Post-Isolation Stroke Prevention and Patient Selection

Thromboembolic risk requires particular attention after LAA electrical isolation because this procedure is associated with a loss of appendage contractility, which in turn promotes blood stasis and thrombus formation. Anticoagulation after ablation is guided by thromboembolic risk rather than by apparent procedural success in restoring sinus rhythm [44]. After LAA electrical isolation, continued OAC or LAA closure is generally required, as thromboembolic events occur predominantly in patients in whom anticoagulation is interrupted or subtherapeutic [3]. More broadly, LAA closure is considered in patients at increased thromboembolic risk who have absolute or relative contraindications to long-term OAC, or in those undergoing LAA isolation who cannot or do not wish to remain anticoagulated [22]. A high HAS-BLED score is not by itself a reason to withhold anticoagulation.

### Hybrid Approaches and Surveillance

In persistent AF, where the LAA may function simultaneously as an arrhythmogenic driver and a thrombus reservoir, combined isolation and closure could offer a potential therapeutic approach. This hybrid strategy could address both rhythm control and stroke prevention.

Following closure, surveillance TEE at 45 days evaluates device position, excludes device-related thrombus, and assesses peri-device flow. Residual peri-device leak is detected in a substantial minority of patients, and its reported frequency varies with the imaging modality used, whereas large leaks (> 5 mm) are uncommon [22, 44, 45].

## LAA-Targeted Management: Clinical Integration and Decision-Making

**Fig. 2:**
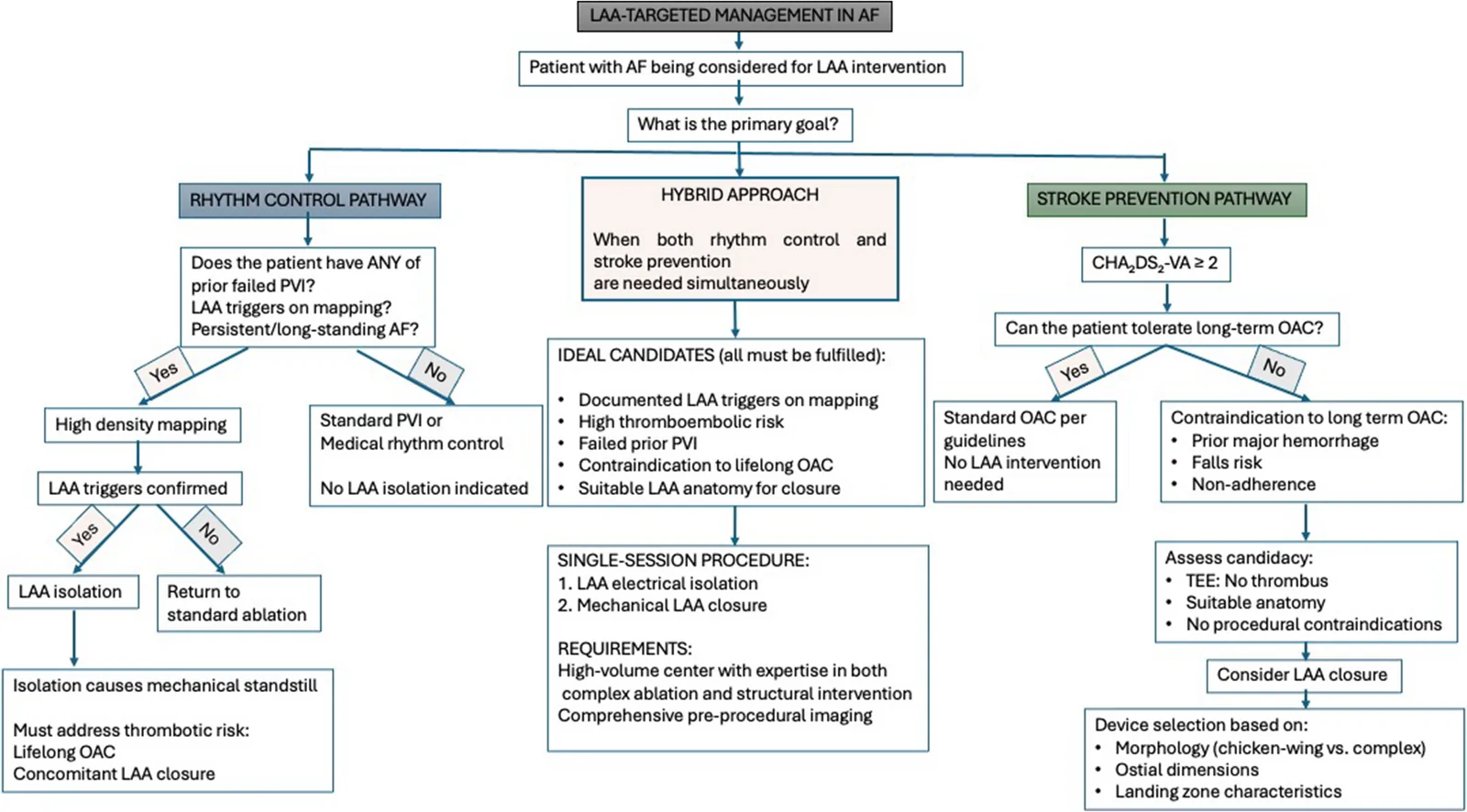
Clinical decision algorithm for LAA-targeted management in atrial fibrillation: electrical isolation may cause mechanical standstill, mandating uninterrupted OAC or concomitant closure to prevent thrombosis; device selection for closure depends on morphology (chicken-wing vs. cactus/windsock/cauliflower) and ostial dimensions determined by pre-procedural imaging; the hybrid pathway requires high-volume centres with expertise in both complex ablation and structural intervention. Key: AF atrial fibrillation, CT computed tomography, ICE intracardiac echocardiography, OAC oral anticoagulation, PVI pulmonary vein isolation

The LAA has a unique position in AF management strategies, being at the same time an arrhythmogenic substrate and a thromboembolic source. Contemporary AF management often requires integrated decision-making that balances these complementary goals of arrhythmia suppression and thromboembolic risk reduction [30]. An algorithmic framework for this process is presented in Fig. 2.

Three pathways emerge: one that focuses on rhythm control, another one on stroke prevention, and a hybrid pathway that integrates the other two approaches. Regarding the first pathway, electrical isolation of the LAA may be considered when high-density mapping identifies LAA-origin triggers in arrhythmia persisting after pulmonary vein isolation and requires circumferential ostial ablation with demonstrated entrance and exit block. Because the resulting mechanical standstill abolishes contractility, it creates the need for uninterrupted anticoagulation or concomitant closure [2, 46]. Mechanical closure addresses a distinct population: those with elevated thromboembolic risk who cannot sustain long-term anticoagulation [30].

Hybrid procedures combining isolation and closure in a single session suit patients with documented LAA triggers, high thromboembolic risk, and either contraindications to or strong preference against lifelong anticoagulation [30, 47]. In summary, optimal AF management incorporates morphological and functional assessment, and patient-specific factors, including bleeding risk and AF burden [22, 30]. Different combinations of these factors may lead to different therapeutic approaches. For example, a patient with paroxysmal AF who has low thromboembolic risk and no documented LAA triggers may require no appendage-specific therapy. A different patient this time with persistent AF, recurrent bleeding on anticoagulation, and mapping-proven LAA triggers may be a suitable candidate for a combined electrical isolation and mechanical closure strategy. Many patients, however, fall between these scenarios and require an individualized approach [30].

## LAA Remodeling in Atrial Fibrillation: Structural-Functional Integration

The LAA does not remodel in isolation. Its structural and functional changes in AF reflect the broader process of atrial and cardiac remodeling.

### LAA Remodeling Correlates with Clinical Risk Scores

The remodeling of the LAA in AF patients as reflected by larger LAA ostial dimensions, lower emptying velocities, and impaired longitudinal strain, was shown to be associated with higher CHA₂DS₂-VASc scores [48, 49]. These findings suggest that CHA₂DS₂-VASc may reflect not only the burden of clinical comorbidities but also the underlying atrial substrate. Higher CHA₂DS₂-VASc scores are also associated with a greater prevalence of LAA thrombus [48–50].

Similarly, markers of a thrombogenic LAA milieu increase with higher CHADS₂ and CHA₂DS₂-VASc scores and are associated with greater stroke risk in patients with AF [51, 52].

### Atrial and Ventricular Interactions

Remodeling of the LAA in patients with AF is tightly connected to the remodeling of the LA and LV. Dilatation of the LA correlates with greater LAA orifice dimensions [10]. Patients with persistent AF demonstrate larger LA volumes than those with paroxysmal AF, and this size difference extends to LAA morphology [10, 53]. The presence of LV diastolic dysfunction further accentuates LAA impairment. Elevated LV filling pressures (reflected by increased E/e′ ratio) associate with reduced LAA flow velocities [54]. In a prospective study using invasive pressure measurement, elevated mean left atrial pressure was independently associated with LAA spontaneous echo contrast, a thrombus precursor, after adjustment for CHA₂DS₂-VASc score and AF type, and LAA peak emptying velocity correlated inversely with mean LAP [55]. Elevated filling pressures may therefore contribute to LAA thrombotic risk in conditions such as HFpEF [54, 55].

Patient-specific factors beyond the CHA₂DS₂-VASc score and remodeling of the neighboring LA and LV may also influence LAA remodeling and hemodynamics in AF. Computational fluid dynamics has recently emerged as a tool for more detailed assessment of LAA blood flow and stasis. Parameters derived from computational methods such as mean blood residence time and LAA anatomical complexity are highly variable among patients with similar CHA₂DS₂-VASc scores, suggesting that they may provide information beyond conventional clinical risk assessment [56]. Further studies showed that LAA hemodynamics and blood stasis are influenced by subject-specific factors, including LA and LAA geometry, cardiac output, and hematocrit [57].

### Systemic Inflammation and LAA Function

Inflammatory markers are also associated with impaired LAA function. An elevated neutrophil-to-lymphocyte ratio (NLR) was associated with reduced LAA wall-motion velocity and spontaneous echo contrast, but not with measures of LA body function. An NLR threshold of 2.5 had 71% sensitivity and 74% specificity for cerebral embolism [58]. More broadly, C-reactive protein levels are elevated in AF and increase with greater AF burden, supporting an association between systemic inflammation and the persistence of the arrhythmia [59].

These results suggest that systemic inflammation may be linked to LAA function and arrhythmia burden in patients with AF.

## Limitations of Current Knowledge

Despite recent advances, there are still key gaps in our knowledge on the LAA role in AF and stroke.

Classifications of the LAA based on morphology, such as “chicken-wing” or “windsock” are subjective and have limited reproducibility. The relationship between LAA structure, contractility, and thrombosis is incompletely understood. There are limited data in the literature relating anatomical or functional features to stroke risk beyond conventional clinical scores. The electrophysiology of the LAA is also incompletely understood. The long-term benefits of LAA electrical isolation or ablation remain uncertain. The clinical value of LAA assessment by computational modeling remains uncertain, as this approach has not yet been sufficiently validated in prospective studies or incorporated into routine clinical practice [60].

## Future Directions and Perspectives

Our knowledge about the LAA in AF is changing rapidly, driven in part by advances in imaging and computational approaches. High-density electroanatomic mapping could be better integrated with imaging to guide ablation and closure procedures [61]. Intracardiac echocardiography and CT can already generate three-dimensional printed models that could in the future improve planning of transseptal access and device placement [62]. Artificial intelligence and computational modeling are being used for prediction of thromboembolic risk, and this could be integrated with other risk prediction modalities [63].

Machine-learning methods are being tested to automatically identify and analyze the different LAA morphology patterns on CT images [64].

Given the limited predictive value of current scores for thromboembolic risk prediction, one path for future research would be to integrate imaging, clinical variables, and inflammatory biomarkers to improve risk prediction, beyond CHA₂DS₂-VASc scoring. Advanced computer models can already estimate blood flow and stasis within the LAA from patient-specific anatomy. These methods could lead to the development of digital-twin models, in which imaging, blood-flow data, and electrical information are combined to simulate the effects of LAA isolation or closure before treatment [65].

Future studies should validate these approaches for patient-specific LAA assessment and treatment planning in clinical practice.

## Conclusions

The LAA is a structurally and functionally complex component of the left atrium with important roles in both AF and stroke. The anatomy and function of the LAA have an impact on thrombus formation, while its electrical properties can contribute to AF. A better understanding of these features may improve the selection of patients for LAA-targeted rhythm-control and stroke-prevention strategies.

## Key References

- Wazni OM, Saliba WI, Nair DG, Marijon E, Schmidt B, Hounshell T, et al. Left Atrial Appendage Closure after Ablation for Atrial Fibrillation. N Engl J Med. 2025;392(13):1277–87.
  - ○ The first randomized comparison of left atrial appendage closure against contemporary oral anticoagulation in patients undergoing AF ablation, establishing non-inferiority for death, stroke, or systemic embolism and superiority for non–procedure-related bleeding.
- Van Gelder IC, Kotecha D, Rienstra M, Bunting KV, Casado-Arroyo R, Caso V, et al. 2024 ESC Guidelines for the management of atrial fibrillation developed in collaboration with the European Association for Cardio-Thoracic Surgery (EACTS). Eur Heart J. 2024;45(36):3314–414.
  - ○ The current European guideline, which reframes stroke-risk assessment around the sex-neutral CHA₂DS₂-VA score within the AF-CARE pathway and defines contemporary indications for appendage-targeted therapy.

## Data Availability

No datasets were generated or analysed during the current study.

## Declarations

### Use of Artificial Intelligence

The authors did not use AI tools in preparing the manuscript, other than AI-assisted copyediting.

## Abbreviations

AF: atrial fibrillation
CFD: computational fluid dynamics
CMR: cardiovascular magnetic resonance
CT: computed tomography
HFpEF: heart failure with preserved ejection fraction
ICE: intracardiac echocardiography
LA: left atrium
LAA: left atrial appendage
LAP: left atrial pressure
LSPV: left superior pulmonary vein
LV: left ventricle
MDCT: multidetector computed tomography
MV: mitral valve
MRI: magnetic resonance imaging
NLR: neutrophil-to-lymphocyte ratio
OAC: oral anticoagulation
PVI: pulmonary vein isolation
PVs: pulmonary veins
TEE: transesophageal echocardiography
